# Influenza vs. COVID-19: Comparison of Clinical Characteristics and Outcomes in Pediatric Patients in Mexico City

**DOI:** 10.3389/fped.2021.676611

**Published:** 2021-06-24

**Authors:** Almudena Laris-González, Martha Avilés-Robles, Clemen Domínguez-Barrera, Israel Parra-Ortega, José Luis Sánchez-Huerta, Karla Ojeda-Diezbarroso, Sergio Bonilla-Pellegrini, Víctor Olivar-López, Adrián Chávez-López, Rodolfo Jiménez-Juárez

**Affiliations:** ^1^Department of Infectious Diseases, Hospital Infantil de México Federico Gómez, Mexico City, Mexico; ^2^Department of Clinical Laboratory, Hospital Infantil de México Federico Gómez, Mexico City, Mexico; ^3^Department of Emergency Medicine, Hospital Infantil de México Federico Gómez, Mexico City, Mexico; ^4^Department of Pediatric Intensive Care, Hospital Infantil de México Federico Gómez, Mexico City, Mexico; ^5^Department of Pediatrics, Hospital de Infectología “Daniel Méndez Hernández,” Unidad Médica de Alta Especialidad Centro Médico Nacional La Raza, Mexico City, Mexico

**Keywords:** influenza, COVID-19, SARS – CoV – 2, children, clinical chracteristics, outcome

## Abstract

**Introduction:** Respiratory viruses are among the leading causes of disease and death among children. Co-circulation of influenza and SARS-CoV2 can lead to diagnostic and management difficulties given the similarities in the clinical picture.

**Methods:** This is a cohort of all children hospitalized with SARS-CoV2 infection from March to September 3rd 2020, and all children admitted with influenza throughout five flu-seasons (2013–2018) at a pediatric referral hospital. Patients with influenza were identified from the clinical laboratory database. All hospitalized patients with confirmed SARS-CoV2 infection were followed-up prospectively.

**Results:** A total of 295 patients with influenza and 133 with SARS-CoV2 infection were included. The median age was 3.7 years for influenza and 5.3 years for SARS-CoV2. Comorbidities were frequent in both groups, but they were more common in patients with influenza (96.6 vs. 82.7%, *p* < 0.001). Fever and cough were the most common clinical manifestations in both groups. Rhinorrhea was present in more than half of children with influenza but was infrequent in those with COVID-19 (53.6 vs. 5.8%, *p* < 0.001). Overall, 6.4% percent of patients with influenza and 7.5% percent of patients with SARS-CoV2 infection died. In-hospital mortality and the need for mechanical ventilation among symptomatic patients were similar between groups in the multivariate analysis.

**Conclusions:** Influenza and COVID-19 have a similar picture in pediatric patients, which makes diagnostic testing necessary for adequate diagnosis and management. Even though most cases of COVID-19 in children are asymptomatic or mild, the risk of death among hospitalized patients with comorbidities may be substantial, especially among infants.

## Introduction

Severe acute respiratory syndrome coronavirus 2 (SARS-CoV2), the virus causing COVID-19, has caused a devastating pandemic with over 100 million cases and 2 million deaths globally so far (1). To date, children have been relatively spared from severe disease and death, representing around 2% of hospitalizations and <1 in every 1,000 COVID-19 deaths (2). However, pediatric patients with underlying conditions have higher rates of hospitalization and death compared with previously healthy children (3).

Other respiratory viral infections such as influenza and respiratory syncytial virus, are among the leading causes of disease and death among pediatric populations (4–7). Co-circulation of influenza and SARS-CoV2 can lead to diagnostic and management difficulties given the similarities in the clinical picture. However, data directly comparing influenza and Covid-19 are scarce, especially in pediatric populations.

The objective of our study was to describe the clinical characteristics and outcomes of children hospitalized with SARS-CoV2 in a pediatric referral hospital and compare them to a cohort of hospitalized children with influenza in the same center.

## Methods

This is a cohort of all children hospitalized with SARS-CoV2 infection from March to September 3rd 2020, and all children admitted with influenza throughout five flu-seasons (2013–2018) at Hospital Infantil de México Federico Gómez, a national referral pediatric hospital with 290 beds and more than 7,000 discharges per year.

Patients with influenza were identified from the clinical laboratory database. Detection of respiratory viruses by PCR multiplex started in 2012 but was established as standard care of children with Severe Acute Respiratory Infection in 2013. We reviewed all records of the subjects with detection of influenza by PCR and included only those that had been hospitalized.

All hospitalized patients with confirmed SARS-CoV2 infection were followed-up prospectively. PCR test was performed on nasopharyngeal swabs of suspect cases beginning in March 2020, and to all admitted patients regardless of symptoms from June 2020.

We collected demographic and clinical variables from the clinical records, including age, sex, comorbidities, symptoms, admission to PICU (pediatric intensive care unit), need for mechanical ventilation and death, among others.

The study was approved by the Research Ethics Committee of Hospital Infantil de México Federico Gómez.

### Microbiology

Nasopharyngeal samples were kept at 2–8°C and processed within 24 h in the SARS-CoV2 cohort and 24 h (up to 72 h in weekends) in the influenza cohort.

In the influenza cohort, we used an RT-PCR system with microarray visualization (CLART PneumoVir, Genomica, Spain) capable of detecting adenovirus, bocavirus, coronavirus, rhinovirus, enterovirus, influenza virus A (subtypes AH3N2, AH1N1), influenza virus B, human metapneumovirus (subtypes A and B), parainfluenza virus 1, 2, 3, and 4 (subtypes A and B), and respiratory syncytial virus type A (RSV-A) and B (RSV-B), with a sensitivity of 83.3–100% depending on the virus. In the case of influenza, the sensitivity and specificity was 91 and 99% for influenza A, 82 and 99% for influenza B, respectively (8).

Two different platforms were used for the detection of SARS-CoV2 throughout the duration of the study: TaqMan^™^ 2019-nCoV Assay Kit v1 (Thermo Fisher Scientific) and 2019 Novel Coronavirus (2019- nCoV) RNA (PCR-Fluorescence Probing) (Da An Gene Co. Ltd. Sun Yat-Sen University). RNA was extracted and purified from all samples using the QIAcube (QIAGEN) system and the QIAamp^®^ Viral RNA mini kit (QIAGEN) following standard manufacturer procedure.

### Statistical Analysis

Categorical data are presented as frequencies and percentages, and quantitative data are provided as median and interquartile range (IQR). Demographic, clinical, and outcome variables were compared between the two groups of patients, using Fisher's exact test for qualitative variables and Mann-Whitney *U*-test for quantitative variables. The comparisons were carried out for all patients and by age group (younger than 1 year, 1–9, and 10 years or older).

We estimated the crude and adjusted odds ratio and 95% confidence intervals (CI) for death and invasive mechanical ventilation using logistic regression. The final model included age a priori; other variables (sex, nutritional status, comorbidities) were included based on reverse stepwise regression (i.e., stopping rule Wald test <0.20). All analyses were performed using STATA 15 statistical package. (College Station, TX, USA: StataCorp).

## Results

A total of 295 patients with influenza and 133 with SARS-CoV2 infection were included, of whom 52 and 55% were male, respectively. The median age was 3.7 years (IQR 1.4–7.7) for influenza and 5.3 years (IRQ 1.1–11.2) for SARS-CoV2. See Table 1 and Supplementary Table 1 for the comparison between the two groups overall and by age group.

**Table 1:** Baseline characteristics of patients with influenza and SARS-CoV2 infection.

	**Total population**			**Symptomatic infection**		
	**Influenza (*n* = 295)**	**SARS-CoV2 (*n* = 133)**	***p*-value**^**†**^	**Influenza (*n* = 293)**	**SARS-CoV2 (*n* = 103)**	**p value**^**†**^
Male sex, *n* (%)	155 (52.5%)	74 (55.6%)	0.60+	139 (47.4%)	46 (44.7%)	0.65
Age, median (IQR)	3.7 (1.4–7-7)	5.3 (1.1–11.2)	0.093	5.3 (1.4–7.8)	7.3 (2–12)	0.002
Any comorbidity	285 (96.6%)	110 (82.7%)	<0.001	283 (96.6%)	85 (82.5%)	<0.001
Cardiovascular	56 (19%)	17 (12.8%)	0.13	55 (18.8%)	13 (12.6%)	0.17
Asthma	17 (5.8%)	5 (3.7%)	0.48	17 (5.8%)	4 (3.9%)	0.61
Pulmonary	42 (14.2%)	5 (3.8%)	0.001	42 (14.3%)	4 (3.9%)	0.004
Immunodeficiency	92 (31.2%)	33 (24.8%)	0.21	92 (31.4%)	25 (24.3%)	0.21
Kidney disease	29 (9.8%)	12 (9%)	0.86	29 (9.9%)	10 (9.7%)	1
Cancer	85 (28.8%)	28 (21%)	0.09	85 (29%)	21 (20.4%)	0.09
Congenital malformations	88 (17.6%)	10 (7.5%)	0.007	52 (17.7%)	9 (8.7%)	0.038
Nutritional status						
Undernourished	88/290(30.3%)	41/131(31.3%)	0.69	87/288 (30.2%)	19/103(28.2%)	0.77
Overweight/Obesity	59/290(20.3%)	22/131(16.8%)	..	59/288 (20.5%)	19/103(18.4%)	..

† *Fisher's exact test or Mann-Whitney test, as appropriate*.

Comorbidities were frequent in both groups, but they were more common in patients with influenza (96.6 vs. 82.7%, *p* < 0.001). Cancer, immunodeficiency states, and heart disease were the most common comorbidities in both groups. Chronic pulmonary disease and congenital malformations were more common among children hospitalized with influenza.

The most frequent indications for hospital admission were pneumonia (46.4% of patients with influenza and 36.8% of patients with SARS-CoV2) and acute decompensation of chronic disease (24.5 and 41.3%, respectively).

Twenty-two percent of patients with SARS-CoV2 had no symptoms attributable to the infection, while only two patients with influenza had subclinical infection. When comparing different age groups, infants were more likely to have asymptomatic SARS-CoV2 infection (48.3 vs. 16.7% among children 1–9 years old, and 13.6% among those 10 years of age and older, *p* = 0.002 by Fisher's exact test).

Among symptomatic children, fever and cough were the most common clinical manifestations in both groups, but cough was more than twice as common in patients with influenza (75.8 vs. 30.1%, *p* < 0.001). Rhinorrhea was present in more than half of children with influenza but was infrequent in those with Covid-19 (53.6 vs. 5.8%, *p* < 0.001). On the contrary, vomiting (29.1 vs. 14.3%, *p* 0.002) and oxygen saturation <92% at hospital admission (40.8 vs. 29.3%, *p* = 0.04) occurred more commonly in children with SARS-CoV2 infection, but this difference was only observed among those 10 years and older when age subgroups were analyzed (see Table 2 and Supplementary Table 2).

**Table 2:** Clinical characteristics of patients with influenza and SARS-CoV2 infection.

	**Total population**			**Symptomatic infection**		
	**Influenza (*n* = 295)**	**SARS-CoV2 (*n* = 133)**	***p*-value**^**†**^	**Influenza (*n* = 293)**	**SARS-CoV2 (*n* = 103)**	***p*-value**^**†**^
Symptoms at diagnosis						
Asymptomatic	2 (0.7%)	30 (22.6%)	<0.001	NA	NA	NA
Fever	248 (84.1%)	78 (58.6%)	<0.001	248 (84.6%)	78 (75.7%)	0.051
Cough	222 (75.2%)	31 (23.3%)	<0.001	222 (75.8%)	31 (30.1%)	<0.001
Pharyngodynia	25 (8.5%)	3 (2.3%)	0.018	25 (8.6%)	3 (2.9%)	0.07
Headache	25 (8.5%)	11 (8.3%)	1	25 (8.5%)	11 (10.7%)	0.55
Myalgia	11 (3.7%)	6 (4.5%)	0.79	11 (3.7%)	6 (5.8%)	0.40
Labored breathing	134 (45.4%)	35 (26.3%)	<0.001	134 (45.7%)	35 (34%)	0.05
Rhinorrhea	157 (53.2%)	6 (4.5%)	<0.001	157 (53.6%)	6 (5.8%)	<0.001
Vomiting	42 (14.2%)	30 (22.6%)	0.037	42 (14.3%)	30 (29.1%)	0.002
Diarrhea	31 (10.5%)	18 (13.5%)	0.41	31 (10.6%)	18 (17.5%)	0.08
SaO2 <92% at hospital admission	87 (29.5%)	50 (37.6%)	0.12	86 (29.3%)	42 (40.8%)	0.04
Hypotension	42 (14.2%)	11/130 (8.5%)	0.11	42 (14.3%)	10/100 (10%)	0.31
Nosocomial acquisition	91 (30.8%)	25 (18.8%)	0.010	89 (30.4%)	13 (12.6%)	<0.001

*SaO_2_, Oxygen saturation; NA, Not applicable*.

† *Fisher's exact test or Mann-Whitney test, as appropriate*.

Admission to the intensive care unit was more common in patients with Covid-19, but the need for invasive mechanical ventilation was similar between groups (see Table 3). Overall, 6.4% percent of patients with influenza and 7.5% percent of patients with SARS-CoV2 infection died. When considering different age groups, a fatal outcome occurred in 12.7% of infants, 4.3% of children 1–9 years old and 7.7% of those 10 years and older with influenza (*p* = 0.08 for the difference between age groups). The rate was 10.3, 6.7, and 6.8% in the same age groups among patients with SARS-CoV2 infection (*p* = 0.83 for the difference between age groups).

**Table 3:** Main outcomes of patients with influenza and SARS-CoV2 infection.

	**Total population**			**Symptomatic infection**		
	**Influenza (*n* = 295)**	**SARS-CoV2 (*n* = 133)**	***p*-value**^**†**^	**Influenza (*n* = 293)**	**SARS-CoV2 (*n* = 103)**	***p*-value**^**†**^
PICU admission	55 (18.6%)	45 (33.8%)	0.001	54 (18.4%)	34 (33%)	0.004
Non-invasive mechanical ventilation	127 (43%)	12 (9%)	<0.001	126 (43%)	10 (9.7%)	<0.001
Invasive mechanical ventilation	67 (22.7%)	27 (20.3%)	0.62	67 (22.9%)	22 (21.4%)	0.79
High-frequency oscillatory ventilation	12 (4.1%)	3 (2.3%)	0.41	12 (4.1%)	2 (1.9%)	0.53
Severe ARDS (PaO2/FiO2 index <100)	19 (6.4%)	3 (2.3%)	0.096	19 (6.4%)	3 (2.9%)	0.01
Death	19 (6.4%)	10 (7.5%)	0.68	19 (6.5%)	9 (8.7%)	0.50

*ARDS, Acute respiratory distress syndrome; PaO2, Partial pressure of oxygen; FiO2, Fraction of inspired oxygen*.

† *Fisher's exact test or Mann-Whitney test, as appropriate*.

In-hospital mortality among symptomatic patients was similar between groups in the multivariate analysis, with an odds ratio of death of 1.87 (0.78–4.45, *p* = 0.16) among patients with Covid-19 as compared to those with influenza after adjusting for age group (<1, 1–9, >10 years), pulmonary disease and malignant neoplasia (see Table 4 and Supplementary Table 2). Age 1–9 years was associated with lower odds of death (OR 0.27, 95% CI 0.19–0.75, *p* = 0.01) in the logistic regression model for the whole population, while a diagnosis of cancer conveyed higher odds of in-hospital mortality (OR 2.72, 95% CI 1.06–6.95, *p* = 0.04).

**Table 4:** Crude and adjusted estimates of the odds ratio for in-hospital death and mechanical ventilation estimated by logistic regression among patients with symptomatic SARS-CoV2 infection in comparison to patients with symptomatic influenza.

	**Outcome (%)**	**Crude OR**	**Adjusted OR**	***p*-value**
Death				
Influenza (*N* = 293)	19 (6.5%)	1.38 (0.60–3.16)	1.87 (0.78–4.45)^†^	0.16
SARS-CoV2 (*N* = 103)	9 (8.7%)			
Mechanical ventilation				
Influenza (*N* = 293)	67 (22.9%)	0.92 (0.53–1.58)	0.83 (0.46–1.49)^‡^	0.53
SARS-CoV2 (*N* = 103)	22 (21.4%)			

† *Adjusted for age group (<1, 1–10, >10 years), pulmonary disease and malignant neoplasia*.

‡ *Adjusted for age group (<1, 1–10, >10 years), pulmonary disease and immunodeficiency*.

The same was true for the need for invasive mechanical ventilation, with an odds ratio of 0.86 (0.49–1.51) among patients with Covid-19 as compared to those with symptomatic influenza, after accounting for age group, pulmonary disease, and immunodeficiency (see Supplementary Table 3).

## Discussion

Respiratory viruses are among the leading causes of pediatric morbidity and mortality worldwide. Unlike other viral respiratory diseases, COVID-19 has had a relatively limited impact on children as compared to adults (9). However, as lockdowns are eased and schools reopened, the increased contact between unvaccinated children might change the scenario and increase the circulation of both SARS-CoV2 and other respiratory viruses, including influenza (10).

In this cohort comparing the clinical characteristics and outcomes of SARS-CoV2 infection with epidemic influenza in hospitalized pediatric patients in a single referral hospital, we found a higher proportion of asymptomatic infection in children with SARS-CoV2 than in those with influenza. Almost half of infants with SARS-CoV2 infection had no symptoms of COVID-19. However, the greater proportion of asymptomatic infection may be explained by the different sampling approach, with PCR testing performed on all admissions beginning in June.

Fever and cough were the most common manifestations among both groups, but rhinorrhea was rare in patients with COVID-19 while it was common in children with influenza. The absence of nasal symptoms might be suggestive of COVID-19 in pediatric patients with fever and cough. However, most signs and symptoms were similar between the two groups, making clinical distinction unreliable.

In comparison, in a retrospective cohort of 315 pediatric patients with COVID-19 (median age 8.3 years) and 1,402 with influenza (median age 3.9 years) in the Unites States, Song et al. (11) describe a higher frequency of fever, gastrointestinal symptoms, headache, myalgia, and chest pain among hospitalized children with COVID-19. However, patients with COVID-19 were older than those with influenza (median age 8.3 vs. 3.9 years) which might have biased the reporting of symptoms.

Another retrospective single-center cohort study found a lower Charlson index in the COVID-19 group, as well as a greater frequency of anosmia, dysgeusia, diarrhea and frontal headache, and lower prevalence of dyspnea, conjunctivitis and vomiting. No children were included in this cohort (12).

Regarding outcome, hospitalized patients with symptomatic influenza and COVID-19 had a similar risk of invasive mechanical ventilation and death, and one in 10 infants had a fatal outcome. This underscores that even though COVID-19 is usually mild in children, it can cause severe disease and death in children with chronic diseases, as has been recognized for influenza for a long time (4). Mortality was lowest among children 1–9 years old with either influenza or SARS-CoV2 infection, but differences between age groups were not statistically significant. This is consistent with reports from the United States, where the greatest proportion of COVID-19 pediatric deaths occurred in infants and adolescents (13) and deaths from influenza are more frequent among those younger than 6 months (14).

The aforementioned study by Song et al. (11) reported a similar rate of hospitalization, intensive care unit admission and use of mechanical ventilation. Two deaths were reported among patients with influenza while no patients with COVID-19 died.

In France, Piroth et al. (15) describe a higher in-hospital mortality for patients with COVID-19 in comparison with those with influenza, based in a nationwide retrospective cohort of hospitalized patients. The proportion of pediatric patients was smaller for COVID-19 than for influenza, but in-hospital mortality was ten-times greater for COVID-19 in those 11–17 years old, and a larger proportion of patients younger than 5 years needed intensive care support for COVID-19 than for influenza.

A systematic review and meta-analysis of studies describing individuals with either influenza or COVID-19 reported a lower frequency of nasal symptoms, pharyngodynia and dyspnea, and a greater prevalence of radiographic abnormalities among patients with COVID-19. Case fatality rate of hospitalized patients was 6.5, 6, and 3%, respectively for individuals with COVID-19, influenza A and influenza B. However, pediatric patients represented a small fraction of the cases and findings were limited by the heterogeneity of the studies (16).

Zhang et al. (17) describe two cohorts of patients hospitalized with influenza or COVID-19 in two separate locations. One in every five patients with COVID-19 was admitted to ICU and 13% died, while no severe or fatal cases were recorded in the influenza cohort. No data for pediatric patients was reported in this study.

Our study has several limitations worth noting. The single-center nature of the cohort, as well as the frequency of comorbidities among participants, may limit the generalizability of findings. Data on patients with influenza was collected retrospectively from clinical records while the COVID-19 cohort was followed-up prospectively. The different time periods between the two cohorts may account from differences in diagnostic and therapeutic approaches. As mentioned above, all patients admitted to hospital were screened for SARS-CoV2 infection from June 2020, which explains the greater proportions of asymptomatic infections. However, asymptomatic patients were excluded from the outcome analysis to account for this limitation. Non-invasive mechanical ventilation was discouraged for patients with suspected or confirmed COVID-19 to reduce aerosol generation, which might have increased the proportion of patients receiving invasive mechanical ventilation.

In conclusion, influenza and COVID-19 have a similar picture in pediatric patients, which makes diagnostic testing necessary for adequate diagnosis and management and will add to the challenge of co-circulation as SARS-CoV2 becomes endemic. Even though most cases of COVID-19 in children are asymptomatic or mild, the risk of death among hospitalized children with comorbidities is substantial, especially among infants, and is similar to that of patients with influenza. Thus, children should not be left out of preventive and therapeutic development in the COVID-19 pandemic, including vaccine development programs (18).

## Data Availability Statement

The raw data supporting the conclusions of this article will be made available by the authors, without undue reservation.

## Ethics Statement

The studies involving human participants were reviewed and approved by Comité de Ética en Investigación del Hospital Infantil de México Federico Gómez. Written informed consent from the participants' legal guardian/next of kin was not required to participate in this study in accordance with the national legislation and the institutional requirements.

## Author Contributions

RJ-J, MA-R, and AL-G contributed to the conception of the study. RJ-J, MA-R, AL-G, IP-O, and JS-H contributed to the design and methodology. CD-B, VO-L, SB-P, KO-D, and AC-L contributed to the acquisition of data. AL-G and RJ-J contributed to the analysis and/or interpretation of data. AL-G drafted the manuscript. All authors contributed to the critical revision of the manuscript for important intellectual content, and approved the final version of this manuscript.

## Conflict of Interest

The authors declare that the research was conducted in the absence of any commercial or financial relationships that could be construed as a potential conflict of interest.
