# Supplementary material for: Influenza vs. COVID-19: Comparison of Clinical Characteristics and Outcomes in Pediatric Patients in Mexico City
Source: Front Pediatr. 2021 Jun 24;9:676611. doi: 10.3389/fped.2021.676611 (PMC8264261; doi:10.3389/fped.2021.676611)
Supplement: Supplementary file 1 [file Data_Sheet_1.PDF]

Supplementary Material

Supplementary Table 1. Clinical characteristics and outcome of patients with influenza and SARS-CoV2 infection by age group.

	< 1 year old			1 to 9 years old			≥ 10 years old		
	Influenza (n=55)	SARS-CoV2 (n=29)	P-value	Influenza (n=188)	SARS-CoV2 (n=60)	P-value	Influenza (n=52)	SARS-CoV2 (n=44)	P-value
Male sex, n(%)	33 (60%)	11 (37.9%)	0.07	99 (52.7%)	40 (66.7%)	0.07	23 (44.2%)	23 (52.3%)	0.54
Comorbidities	53 (96.4%)	24 (82.8%)	0.045	181 (96.3%)	48 (80%)	<0.001	51 (98.1%)	38 (86.4%)	0.045
Cardiovascular	27 (49.1%)	7 (24.1%)	0.04	29 (15.4%)	9 (15%)	1	1	1 (2.3%)	0.46
Pulmonary	13 (23.6%)	3 (10.3%)	0.24	25 (13.3%)	2 (3.3%)	0.03	4 (7.7%)	0	0.12
Immune deficiency	4 (7.3%)	0	0.29	59 (31.4%)	21 (35%)	0.64	29 (55.8%)	12 (27.3%)	0.007
Kidney disease	2 (3.6%)	1 (3.4%)	1	18 (9.6%)	5 (8.3%)	1	9 (17.3%)	6 (13.6%)	0.78
Cancer	2 (3.6%)	0	0.54	58 (30.8%)	17 (28.3%)	0.75	25 (48.1%)	11 (25%)	0.0
Congenital malformations	18 (32.7%)	0	<0.001	33 (17.5%)	6 (10%)	0.22	1 (1.9%)	4 (9.1%)	0.18
Symptoms at diagnosis									
Asymptomatic	2 (3.6%)	14 (48.3%)	<0.001	0	10 (16.7%)	<0.001	0	6 (13.6%)	0.008
Fever	41 (74.5%)	7 (24.1%)	<0.001	163 (86.7%)	39 (65%)	<0.001	44 (84.6%)	32 (72.7%)	0.21
Cough	35 (63.6%)	5 (17.2%)	<0.001	150 (79.8%)	13 (21.7%)	<0.001	37 (71.1%)	13 (29.5%)	<0.001
Pharyngodinia	NA	NA	..	15 (8%)	0	0.02	9 (17.6%)	3 (6.8%)	0.13
Headache	NA	NA	..	13 (6.9%)	3 (5%)	0.77	11 (21.1%)	8 (18.2%)	0.8
Laboured breathing	33 (60%)	8 (27.6%)	0.006	90 (47.9%)	11 (18.3%)	<0.001	11 (21.1%)	16 (36.4%)	0.11
Rhinorrhea	21 (38.2%)	0	<0.001	107 (56.9%)	4 (6.7%)	<0.001	29 (55.8%)	2 (4.5%)	<0.001
Vomiting	11 (20%)	5 (17.2%)	1	27 (14.4%)	13 (21.7%)	0.22	4 (7.7%)	12 (27.3%)	0.01
Diarrhea	8 (14.5%)	0	0.046	21 (11.2%)	13 (21.7%)	0.05	2 (3.8)	5 (11.4%)	0.24
SaO2 <92% at hospital admission	21 (38.2%)	12 (41.2%)	0.82	55 (29.3%)	18 (30%)	1	11 (21.1%)	20 (45.4%)	0.016

Supplementary Table 1 (continued). Clinical characteristics and outcome of patients with influenza and SARS-CoV2 infection by age group.

Hypotension	9 (16.4%)	2 (6.9%)	0.32	22 (11.7%)	4 (6.8%)	0.34	11 (21.1%)	5 (11.9%)	0.28
Nosocomial acquisition	28 (50.9%)	12 (41.4%)	0.49	49 (26.1%)	4 (6.7%)	0.001	14 (26.9%)	9 (20.4%)	0.48
Outcome									
PICU admission	17 (30.9%)	17 (58.6%)	0.02	28 (14.9%)	12 (20%)	0.42	10 (19.2%)	16 (36.4%)	0.07
NIMV	24 (43.6%)	3 (10.3%)	0.003	80 (42.5%)	4 (6.7%)	<0.001	23 (44.2%)	5 (11.4%)	0.001
IMV	19 (34.5%)	7 (24.1%)	0.46	37 (19.7%)	8 (13.3%)	0.34	11 (21.1%)	12 (27.3%)	0.63
HFOV	4 (7.3%)	2 (6.9%)	1	7 (3.7%)	1 (1.7%)	0.68	1 (1.9%)	0	1
PaO2/FiO2 index <100	5 (9.1%)	0	0.16	8 (4.3%)	0	0.12	5 (9.6%)	3 (6.8%)	0.72
Death	7 (12.7%)	3 (10.3%)	1	8 (4.3%)	4 (6.7%)	0.49	4 (7.7%)	3 (6.8%)	1

SaO2: Oxygen saturation, NIV: Non-invasive mechanical ventilation, IMV: Invasive mechanical ventilation, HFOV: High-frequency oscillatory ventilation.

PaO2: Partial pressure of oxygen, FiO2: Fraction of inspired oxygen, NA: Not applicable.

Supplementary table 2. Results of the logistic regression model for in-hospital mortality among patients with symptomatic SARS-CoV2 infection in comparison to patients with symptomatic influenza (baseline).

**Variable**	**Odds Ratio**	**95% CI**	**p value**
SARS-CoV2 vs influenza	1.77	0.73-4.29	0.15
Age group			
< 1 year	Reference		
1 to 9 years	0.27	0.19-0.75	0.01
≥10 years	0.41	0.12-1.32	0.13
Sex	0.57	0.25-1.30	0.18
Pulmonary disease	2.58	0.89-7.46	0.08
Cancer	2.72	1.06-6.95	0.04

CI: Confidence interval.

Supplementary table 3. Results of the logistic regression model for invasive mechanical ventilation among patients with symptomatic SARS-CoV2 infection in comparison to patients with symptomatic influenza (baseline).

	Odds Ratio	95% CI	p value
SARS-CoV2 vs influenza	0.83	0.46-1.49	0.53
Age group			
< 1 year	Reference		
1 to 9 years	0.60	0.32-1.11	0.10
≥10 years	1.10	0.52-2.33	0.80
Pulmonary disease	1.75	0.88-3.49	0.11
Immunodeficiency	0.42	0.22-0.80	0.008

CI: Confidence interval.
